# Confidence in uncertainty: Error cost and commitment in early speech hypotheses

**DOI:** 10.1371/journal.pone.0201516

**Published:** 2018-08-01

**Authors:** Sebastian Loth, Katharina Jettka, Manuel Giuliani, Stefan Kopp, Jan P. de Ruiter

**Affiliations:** 1 Social Cognitive Systems, CITEC, Bielefeld University, Bielefeld, Germany; 2 Psycholinguistics, CITEC, Bielefeld University, Bielefeld, Germany; 3 Psycholinguistics, Linguistics and Literary Studies, Bielefeld University, Bielefeld, Germany; 4 Bristol Robotics Laboratory, University of the West of England, Bristol, United Kingdom; 5 Social Cognitive Systems, Faculty of Technology, Bielefeld University, Bielefeld, Germany; 6 Departments of Psychology and Computer Science, Tufts University, Medford, Massachusetts, United States of America; Icahn School of Medicine at Mount Sinai, UNITED STATES

## Abstract

Interactions with artificial agents often lack immediacy because agents respond slower than their users expect. Automatic speech recognisers introduce this delay by analysing a user’s utterance only after it has been completed. Early, uncertain hypotheses of incremental speech recognisers can enable artificial agents to respond more timely. However, these hypotheses may change significantly with each update. Therefore, an already initiated action may turn into an error and invoke error cost. We investigated whether humans would use uncertain hypotheses for planning ahead and/or initiating their response. We designed a Ghost-in-the-Machine study in a bar scenario. A human participant controlled a bartending robot and perceived the scene only through its recognisers. The results showed that participants used uncertain hypotheses for selecting the best matching action. This is comparable to computing the utility of dialogue moves. Participants evaluated the available evidence and the error cost of their actions prior to initiating them. If the error cost was low, the participants initiated their response with only suggestive evidence. Otherwise, they waited for additional, more confident hypotheses if they still had time to do so. If there was time pressure but only little evidence, participants grounded their understanding with echo questions. These findings contribute to a psychologically plausible policy for human-robot interaction that enables artificial agents to respond more timely and socially appropriately under uncertainty.

## Introduction

Face-to-face interactions between humans are the model for intuitive and social human-machine interaction (HMI). Humans respond sensibly and swiftly to their interlocutors’ utterances and establish immediacy in the interaction. This exchange of conversational turns systematically minimises silence and overlapping talk [1]. The next speaker typically starts speaking within a small time window ranging from 250 ms before and after the end of an utterance [2]. A preference for very brief periods of silence was found across cultures [3]. Precisely timed responses are conventionalised in human interaction. Thus, temporal misalignments constitute a signal to interlocutors [4], e.g. gaps may signal disagreement [5,6]. In HMI, prolonged system response times slow down turn-taking, reduce the agent’s immediacy, cause annoyance, reduce user motivation [7,8] and users might falsely believe that they made a mistake in how they were communicating with the system. Therefore, to be socially competent, an artificial agent has to respond with precise timing.

Humans achieve timely responses despite the fact that it takes approximately 600 ms for preparing and initiating the articulation of a single word [9–11]. More complex utterances require considerably longer [12]. Thus, understanding an utterance and planning a response occur concurrently for at least 400 ms. This implies that humans understand utterances incrementally [2]. Evidence suggests that they start trying to predict the end of an utterance by as much as 1250 ms [13]. The effects of incrementality are most apparent in mistakes, e.g. garden path sentences typically require a re-analysis [14,15]. Correct predictions enable the listener to estimate their interlocutor’s speech act ahead of time and to prepare a sensible and timely response [16,17]. But the mapping between utterances and speech acts is highly ambiguous. Thus, heuristics are required for identifying the intended speech act [4,18]. Both, predicting an utterance and heuristically estimating the speech act are imperfect processes, introduce uncertainty and might result in costly errors. Humans try to avoid errors with several strategies. First, humans account for larger parts of a discourse [17,19] and use knowledge about the scenario (e.g., scripts, see [20,21]) when forming predictions rather than relying only on local information. Secondly, listeners produce so-called *grounding* signals to communicate their current understanding of an utterance to the speaker, e.g. they gesture [22] or gaze towards relevant objects [23,24] or objects that will become relevant [25,26]. These grounding signals enable speakers to adapt their utterances on-the-fly [27]. Thirdly, humans typically initiate their response after their interlocutors almost completed their utterances [22,28]. This enables them to compare their predictions to the actual utterance and to adapt their responses if needed. These mechanisms enable timely responses and reduce the risk of costly errors in incremental, predictive processing.

From their daily experience of interacting with other humans, users expect that artificial agents respond swiftly, sensibly and provide relevant grounding signals. But artificial agents have to rely on automatic speech recognisers (ASR). Recently, ASR performance exceeded human performance in conversational speech [29] and improved in noisy environments [30]. However, ASRs typically provide a final hypothesis about the speaker’s utterance only after 1.6 s [31] which is outside the +/- 250 ms window in human interaction. Incremental ASRs (ISR) deliver uncertain hypotheses while utterances unfold, e.g., Kaldi [32,33], Sphinx-4 [34], Google Voice API and Microsoft Speech Recognition APIs. Thus, they can enable artificial agents to respond more timely. However, early speech hypotheses are incomplete and uncertain. The hypotheses’ content may change as more material of the unfolding utterance has been analysed. In contrast to humans, artificial agents cannot wait for the final and most certain ASR analysis without sacrificing the temporal advantage of an ISR. For example, a system trained with inverse re-inforcement learning to detect the end of a turn and initiate a speak or backchannel action showed an increase in the probability of responding to over 50% after about 1.0 s of silence [35]. However, a pause of 1.0 s is still large compared to a fluent interaction and does not include the time required by the ASR for recognising a pause. Thus, an artificial agent has to fully commit and initiate its response based on an early and therefore more uncertain speech hypothesis.

Incomplete speech hypotheses have to be projected onto an utterance that is sufficiently informative for initiating an appropriate response. This has been achieved, e.g., by fitting a syntactic structure to a hypothesis, disambiguating syntactic ambiguities with knowledge about the scene and further projecting this onto a collection of fully annotated utterances [36,37]. This way the system guesses the full utterance and its semantic frame [38–40]. The early semantic information can be used for producing grounding signals [38,40]. Vice versa, processing the user’s actions during the artificial agent’s utterance enables the agent to estimate the user’s understanding [41,42], to adapt its utterance and to explicitly elicit user feedback if needed [43–45]. These accounts aim for integrating incremental hypotheses into a larger, multimodal model of intention recognition. The AIRBUS model [16,18] integrates multimodal inputs and prior knowledge with Bayesian updates over the probabilities of a set of intentions. This identifies the most likely speech act and how this deviates from the model’s prior expectations at any moment in time. The architecture can be extended to capture the processing from basic units of computer perception to intentions in hierarchical layers [46,47]. Further improvements to ISRs were achieved by contextually boosting parts of the vocabulary [48] and estimating the end of an utterance, e.g., using morae in Japanese [49]. Furthermore, projecting incomplete onto full utterances magnifies the ISR’s uncertainty and in turn, the risk of a significant revision that requires re-planning increases. Thus, dialogue managers have to balance the risk of costly errors with the benefit of more timely responses. In order to explicitly model uncertainty, dialogue managers shifted from Markov decision processes (MDPs) to partially observable MDPs (POMDPs, see [50,51] for introduction and [52] for review). These systems focussed on the uncertainty after the final analysis of an utterance and its corresponding n-best list of hypotheses in POMDPs [53,54] and recurrent neural nets [55]. However, the large training sets that are required with these models are often derived from simulated users. This limits the model’s performance to the quality of the simulation. Gaussian process policy optimisation reduces the number of required dialogues by a magnitude to only 10,000s of dialogues that are feasible to create with Amazon MTurk [56]. The policy trained with real input significantly outperforms simulator trained policies [56]. By using domain independent knowledge such as syntactic and lexical knowledge, Eshgi et al. [57] were able to demonstrate that as little as 5 dialogues were sufficient for training. Furthermore, their incremental word-by-word processing improved the quality of the dialogues but the incremental parses were based on text that did not change or update during the parse.

The abstract ’incremental unit’ (IU) model [58–61] explicitly addresses incremental speech hypotheses. It maintains a record of each hypothesis but it does not address efficiency and error cost. The incremental interaction manager [62] immediately performs a response action if it advances the dialogue. An ongoing action is stopped if an update triggers a more appropriate action. This model does not require tracing hypotheses but it may commit unnecessary errors. For example, prematurely closing the interaction with the user cannot easily be interrupted or undone. Erroneous closings require the user to start all over again and thus, have high error cost [63,64] whereas other types of actions that incur lower error cost could be triggered immediately. But there is no clear evidence on quantifying error cost and how it interacts with the users’ expectation on immediacy in the interaction.

The research on human-human interactions showed that humans predict utterances and actions of others. Humans use these predictions for planning ahead and for producing grounding signals but often initiate their response only after almost the entire utterance has unfolded. Thus, there is little evidence for designing a psychologically plausible dialogue manager that uses incremental speech hypotheses for initiating response actions and accounts for the respective error cost. We therefore investigated whether humans use incremental speech hypotheses for grounding, planning ahead or for immediately performing actions, and summarised the results in an abstract interaction policy.

## Ghost-in-the-Machine study

We conducted a real-time Ghost-in-the-Machine study (GiM, see [65,66]). The participants controlled a bartending robot that accepted orders from its customers and served the corresponding drinks. In contrast to typical Wizard-of-Oz studies, see [67] for review, the participants cannot observe the scene through a video or audio link but have to rely on preprocessed sensor data including the ISR speech hypotheses. That means that the main participants in our study (ghosts) observed their bar customers through the robot’s eyes and ears and responded to them by selecting actions from the robot’s repertoire. In this study, we focus on their use of incremental speech hypotheses. These were real ISR hypotheses including mistakes induced by the customers (e.g., slips of the tongue) and any recognition mistakes, uncertainties and updates by the ISR.

The ghosts were familiarised with the interfaces of this GiM study. They were explained that the ISR hypotheses were real and subject to uncertainties and errors. But they were not instructed how they should interact with their customers. Thus, their actions approximate how they would use speech hypotheses themselves and by extension, how they expect others to use them. We hypothesised that the ghosts would rely on the early hypotheses for planning a response but not necessarily for initiating it. Furthermore, the evidence from human-human interaction suggests that mechanisms in interaction including turn-taking (i.e., timely responses) obey the same timing constraints independently from the potential error cost associated with a particular response.

We refer to the main participants controlling the robot as *ghosts* and to the confederates placing the orders as *customers*.

### Methods and materials

#### Conditions

In order to test whether the ghosts used early, uncertain speech hypotheses, we designed a *certain* and an *uncertain* condition. The *certain* condition reflected typical dialogue managers or action planners where all data with a confidence level exceeding a threshold are treated as ground truth whereas other data are discarded. Thus, the ghosts were presented only final hypotheses that were displayed with the maximum confidence level. In the *uncertain* condition all available data together with their true confidence level were presented, i.e. the ghosts had access to faster and additional information. A dialogue manager would have to integrate the confidence levels and the content of the hypotheses. By comparing the response times (RT) in *certain* and *uncertain* trials, we studied how the presence of the additional early but uncertain speech hypotheses affected the ghost’s behaviour.

#### Scenario and materials

A typical bar scene involves multiple customers. The bartending robot has to accept drink orders and subsequently serve the ordered drinks to its customers. In addition, the robot has to recognise its customers’ social signals (e.g., the intention to order) and respond intuitively and socially appropriately. We derived six simple drink order scripts from previously recorded empirical data [68]. We selected two examples for each: a) one confederate ordered a drink and the second confederate was a bystander, b) both confederates ordered their drinks individually, and c) one confederate ordered both drinks (group order). In addition to the drink orders, some trials included questions about the menu. In these trials, a customer asked which drinks or whether a particular drink was available prior to placing their order. In order to answer the questions, the ghosts would respond verbally, e.g. by enumerating the menu consisting of three drinks. The drink orders required the physical serving of a drink. Due to technical limitations, this action could not be interrupted and the drink could not be retracted. Thus, an erroneous serving was associated with high error cost. In contrast, customers approaching the robotic bartender might not be familiar with its drinks menu. In this case, listing the menu was appropriate even if the customers did not ask for it. So the error cost was considerably higher for an erroneous drink serving than for an unsolicited enumeration of the drinks menu.

In a typical trial, the ghosts observed how the customers entered the area covered by the sensors and approached the bar. The ghosts initiated an interaction, the customers asked their question and placed their order(s), the robot served the drink(s), the interaction was closed and the customers left the scene with their drinks. All scripts are listed in S1 File.

In our study, the same two confederates acted as customers throughout the experiment. They staged scripted drink order scenarios for the robot (and by implication, for the ghosts controlling the robot). This procedure served to minimise user specific variation of the recognisers, especially the ISR. The assignment of the roles (bystander, group member, placing an order) and the choice of drinks (water, juice, coke) were counterbalanced between the confederates.

#### Participants

Seventeen participants (4 female, 13 male, age range 21–39, M = 28.5, Mdn = 27) were recruited out of the employees of fortiss GmbH in Munich, Germany (all with a background in IT or engineering). The participants were not familiar with the purpose of the study. They received 5 € and a chocolate bar in exchange for their time and effort.

The study was conducted by scientists of Bielefeld University, Germany. Its procedures were approved by Bielefeld University’s Ethics Committee (EUB) under approval №4807. An informed written consent was collected prior to the experiment.

#### Apparatus

The participants were seated at a desk with two typical office screens (52 cm by 32 cm, 1920 by 1200 pixel), mouse and keyboard in a room separating them from the bartending robot. The first screen was positioned straight in front of the participants at a viewing distance of approximately 70 cm. It displayed the recogniser data. The participant’s eye gaze on this screen was recorded using a 60 Hz remote eye tracker [69] positioned below the centre of the screen. Data were recorded on the local computer at 60 Hz. Infrared illuminator pods were positioned on top of the screen, below the screen and in the central position between the cameras. Switching between the pre-installed pods speeded the process of finding a suitable illumination and tracking mode for each participant. The eye tracker could be calibrated to all participants satisfactorily for the purpose of this study.

**Fig 1:**
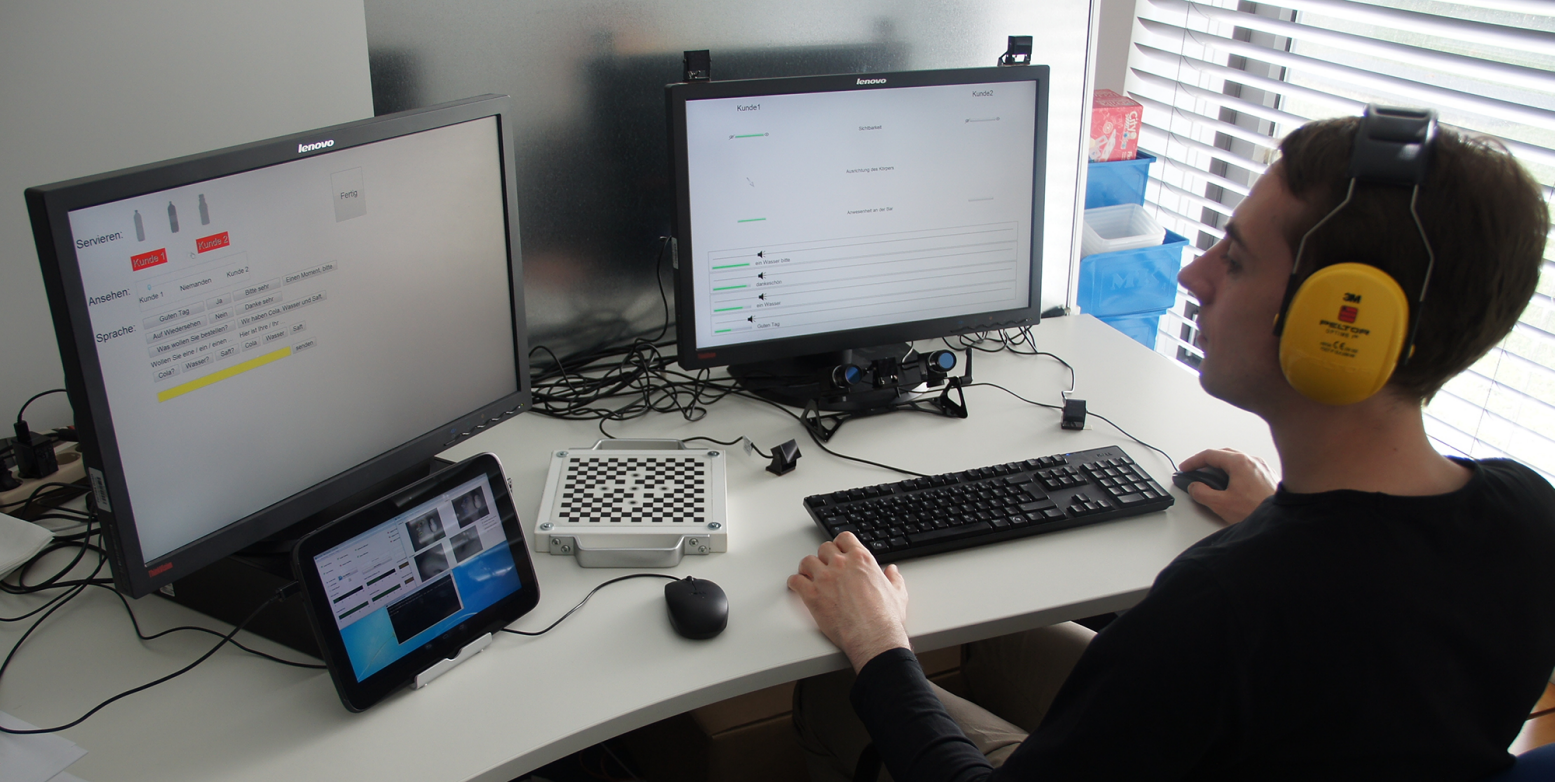
Setting of the study. The setting for the ghost participants including the information panel, eye tracker, control panel and eye tracker control screen. The participant wears passive noise insulating headphones.

The robot’s control interface was presented on the second screen which was positioned to the left hand side of the participants and outside of the eye tracker’s range. If the ghosts wished to control the robot, they had to turn left and away from the screen in front of them. Thus, even with a relatively coarse tracking precision, we were still able to reliably determine when the participants switched to the control panel and which recogniser data had been visually attended. The participants were shielded from distractions by portable blank screens around the desk and passive sound insulating headphones. An experimenter checked the functioning of the eye tracker through a tablet computer connected via WiFi. This experimenter stayed in the room in order to adjust the eye tracker if needed. It was obvious to the participants that they were not monitored directly or through the tablet. The setup is shown in Fig 1.

#### Robot and sensors

**Fig 2:**
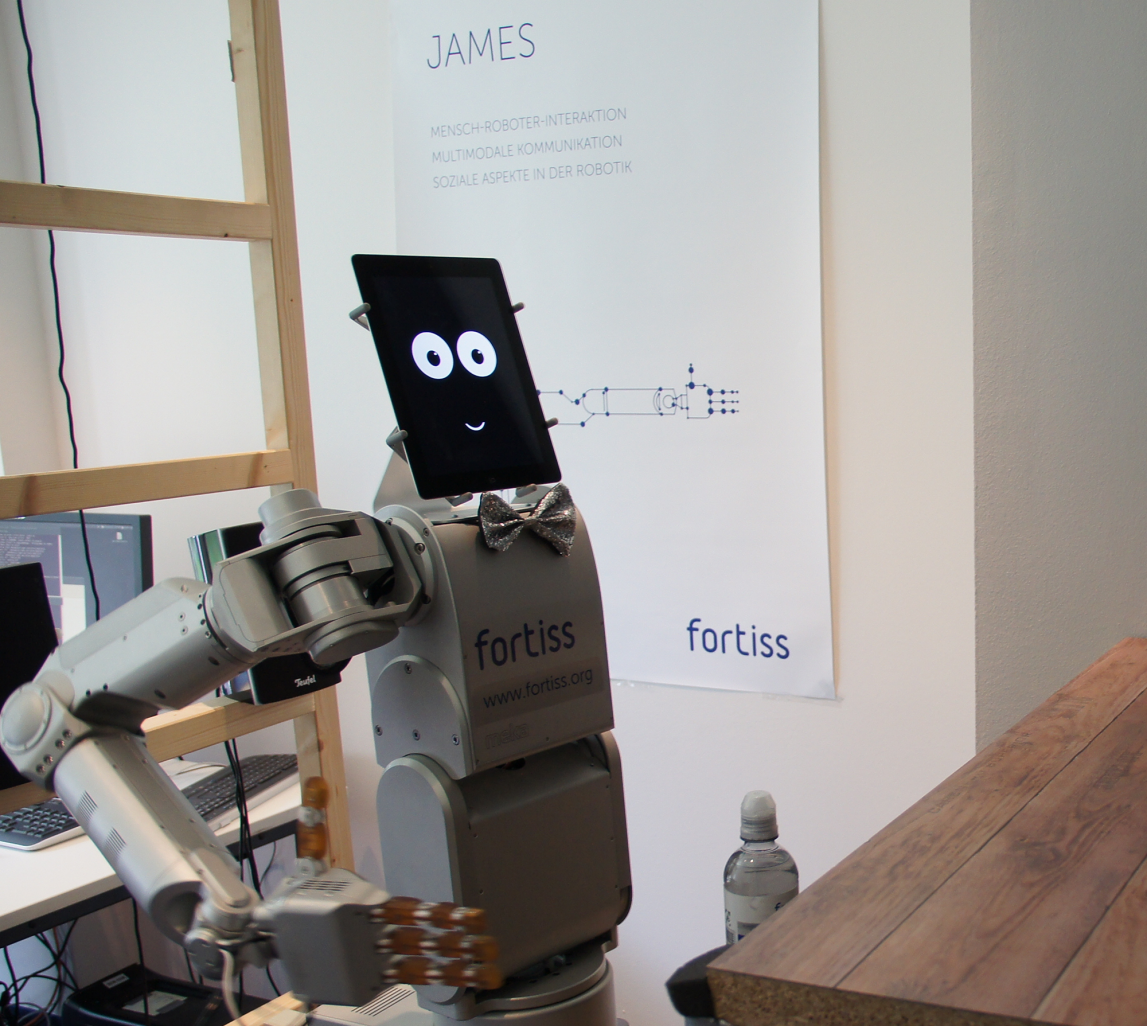
Bartending robot. The robot is shown at its bar about to grab a bottle of water for serving.

The robot (Fig 2) consisted of a Meka torso, right arm and hand and an iPad with a comic style representation of eyes and mouth implemented in HTML5. It served the drinks to the customers and a text-to-speech programme voiced the ghosts’ utterances. The ghosts’ commands were also shown in plain text on the tablet computer next to the face. This provided an additional channel for the customers, e.g. if the text-to-speech was distorted due to typos.

The customers were tracked by a Microsoft Kinect unit. We implemented a tracking module that provided updates about the customers’ visibility, distance to the bar and their torso orientation based on the Kinect’s skeleton data. We implemented a speech recognition module that used the Microsoft speech recognition and implemented a Speech Recognition Grammar Specification (SRGS) containing only sentences related to the bar scenario. This included greetings, drink orders, clarifications, and sentences to conclude the interaction. The Kinect, the robot and the ghost’s interface were connected through a middleware at 20 Hz.

#### Ghost-in-the-Machine interface

**Fig 3:**
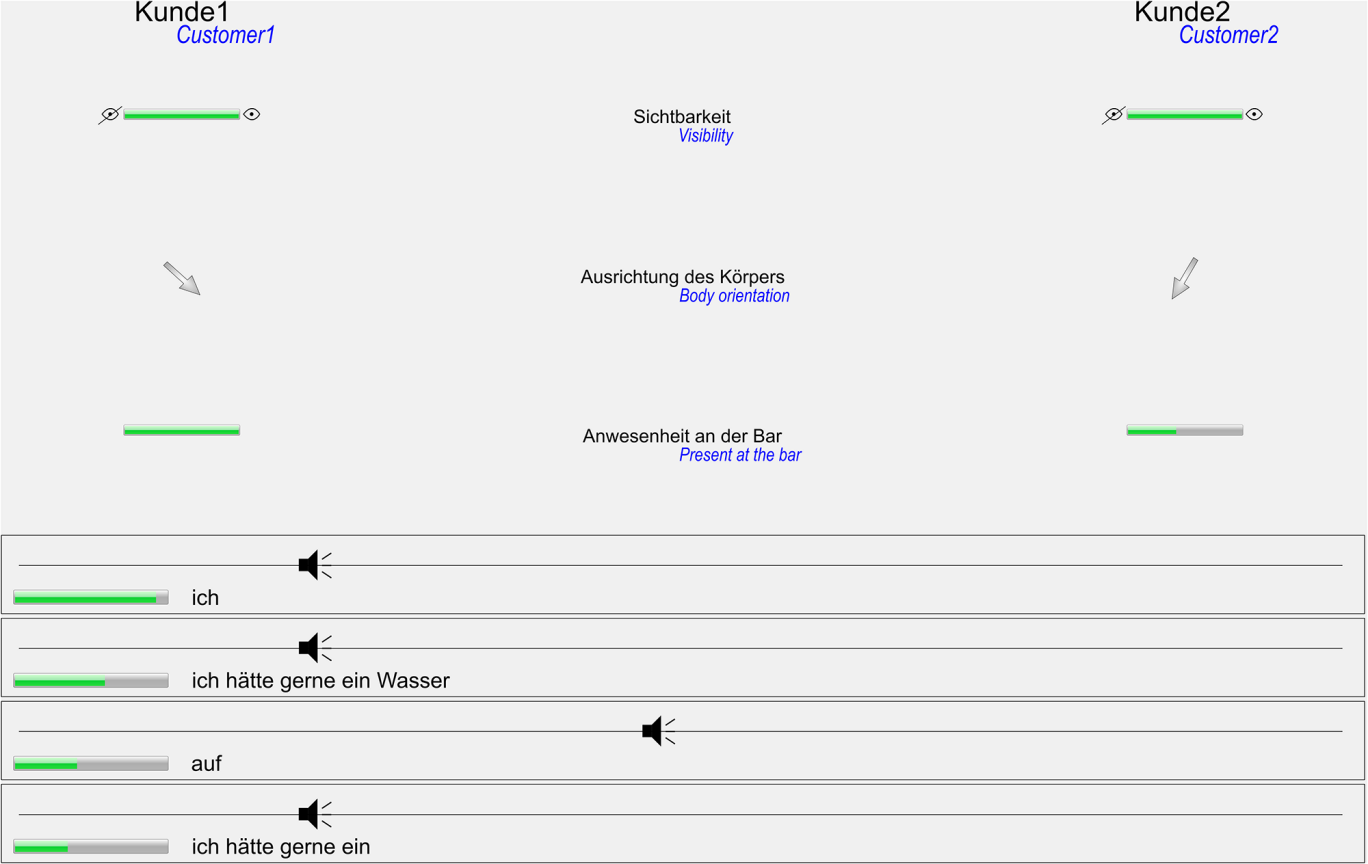
Information panel for the ghosts. The panel covered the entire computer screen in front of the participants. Translations are provided in blue and were not part of the experimental design.

**Fig 4:**
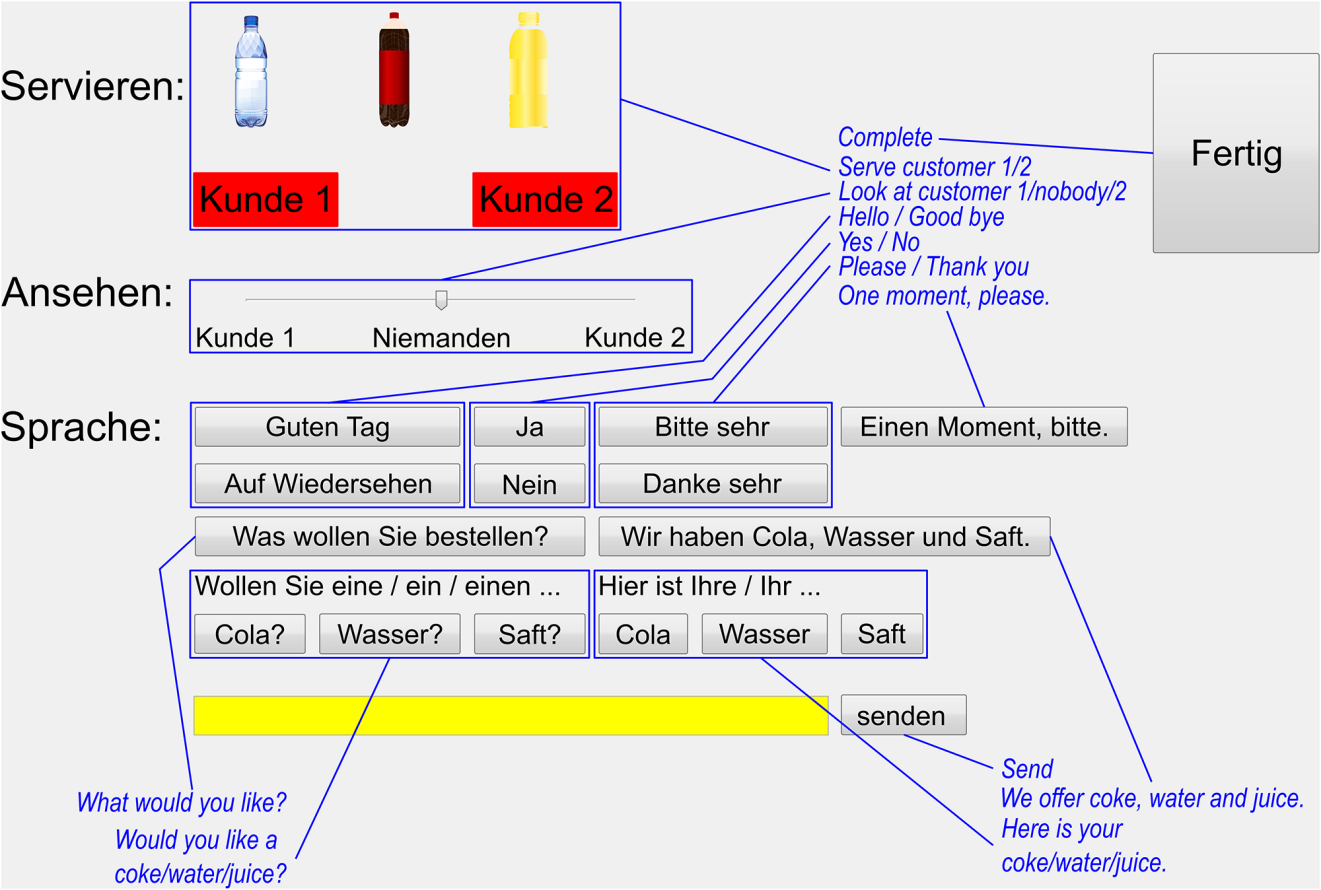
Control panel for the ghosts. The panel was shown in the upper left corner of the screen. The remaining screen was light grey matching the panel’s background colour. Translations are provided in blue and were not part of the experimental design.

The ghosts’ interface was a dedicated JAVA application that managed the flow of information to and from the middleware. It consisted of an information panel that showed the incoming recogniser data (Fig 3). This was displayed on the screen with the eye tracker straight in front of the ghosts. The screen on the left hand side displayed the control panel (Fig 4). Any selected robot actions were executed immediately. The remaining area on either screen was covered by a plain single coloured background. In contrast to previous off-line GiM studies [65,66], this design enabled the ghosts to engage directly with their customers and observe how they responded to their actions.

#### Information panel

The information panel was divided into two larger sections. In order to facilitate the ghosts’ real-time understanding, the upper part was limited to the most relevant information from computer vision [66]: the customers’ visibility, distance and body orientation. In the *uncertain* condition, the progress bar for the customer’s visibility reflected the system’s confidence of having detected a person in the tracking area whereas in the *certain* condition the bar was either empty or full. The other progress bar indicating whether a customer is at the bar. It linearly mapped the customer’s distance between 0.8 and 0.3 m in the *uncertain* condition and a threshold of 0.6 m was used in the *certain* condition for switching between empty and full. The arrow indicator for the body orientation was not affected by the condition. The lower section of the information panel was dedicated to the ISR hypotheses. Up to four hypotheses were displayed with their content as plain text and a progress bar indicating the ISR’s confidence. In addition, the utterance’s source direction as measured by the Kinect’s microphone array was depicted by a speaker symbol on a slider bar and indicated whether customer 1 or 2 had spoken. In the *certain* condition, only final hypotheses were displayed and the progress bar was always set to full. In the *uncertain* condition, all hypotheses were presented with their true confidence value.

#### Time morphing

We presented the ISR data visually because an ergonomical and accurate auditory presentation of incrementally growing content and hypotheses that differ only in their confidence level is (almost) impossible. Due to smoothing and other post-processing ASRs are slow in producing hypotheses compared to a human listener. However, the ISR produces hypotheses faster than humans can read and understand them. Visually understanding the presented speech hypotheses requires explicitly combining their spatial origin, certainty and content whereas these data are inherently combined when listening to an utterance. If the time span between two hypotheses is too short, we cannot distinguish whether the ghosts a) attended a fast stream of incoming information but could not process it, b) perceived the information as still unreliable and waited for more/better data, or c) wished to act but new information appeared and distracted them from doing so. Thus, in order to ensure that we can interpret our results, we extended the time span between hypotheses to a minimum of 1500 ms (see S1 and S2 Videos for comparison).

Displaying hypotheses was managed by entering all new hypotheses into a queue that maintained their temporal order. The next hypothesis in the queue was displayed whenever the most recent hypothesis has been displayed for at least 1500 ms. Up to four hypotheses were presented in descending order of their confidence level. Since later hypotheses tended to be more confident, they typically entered the display at the top position. If the content and direction of a new hypothesis matched with an already displayed item, the confidence level was updated. Some hypotheses entered the temporal queue but were never displayed because their confidence was too low. Old speech hypotheses were removed from the display or the queue after 4000 ms and the next hypothesis was displayed. The display in the *certain* condition was rarely affected by time morphing because each utterance was typically associated with one final hypothesis. The time morphing allowed the ghosts sufficient time for reading the hypotheses but it also slowed the speech recognition process, especially in the *uncertain* condition. For this reason the customers could have perceived the ghosts’ responses as very slow. In order to mitigate this, the customers had an additional display of their own ISR hypotheses and were instructed to wait patiently. None of the ghosts commented on the speed of the ISR nor did they appear to notice our manipulation.

#### Control panel

The control panel (Fig 4) was arranged in three groups: serving, gazing and speaking. Serving a drink was achieved by dragging the respective bottle onto the customer’s serving zone and dropping it. The ghosts shifted the slider bar to either side for gazing at the respective customer or returned it to its neutral position. The slider remained at its position until the ghost changed it or the interface was reset at the end of a trial. The area below was dedicated to speech and included clickable pre-worded utterances. Below the buttons, the ghosts could type into a free text field for triggering an individual utterance. All actions were forwarded to the middleware and executed immediately. The ghosts could combine actions into a complex response by clicking several actions, e.g. by selecting a gaze direction and a speech utterance. However, the interface hindered the ghosts from selecting actions that could not be performed simultaneously, e.g. saying two things at once. This was achieved by locking buttons group-wise for as long as an action was ongoing, e.g. all speech related buttons were locked for as long as the robot was speaking. The ghosts were asked to press the *Complete*-button if they felt they had served their customers and were ready for the next interaction. They were warned that pressing this button too early would abruptly terminate an ongoing interaction.

#### Procedure

The participants (ghosts) were welcomed to the experiment, introduced to the bartending robot, the bar and the available drinks (coke, water and juice). They were shown the office where the experiment was conducted, the equipment and their task. After introducing the setting, the participants were asked to provide their written consent. If they agreed to partake in the study, they attended a presentation, introducing in detail the experiment, their task and how to use the interface. It emphasised that the sensor data were real and possibly unreliable. The eye tracker was calibrated for the screen in front of the participants.

Each trial started with a message on the ghost’s screen indicating that the robot was waiting for new customers. The confederates were presented the script of the trial on their interface at a position outside the robotic sensors’ scope. They started the trial through a wireless keyboard. This cleared the message on the ghost’s display. The customers placed their orders according to the script, took their drinks and returned to their start position. The trial ended as soon as the ghost clicked the *Complete*-button. This triggered the message that the robot was waiting for new customers and informed the confederates about the next trial. In case of any problems, the experimenter in the room and the confederates communicated through an online chat and tried to rectify them.

The experimental session started with two practice trials in fixed order that were excluded from further analyses. In the first practice trial, a single customer approached the robot and ordered a single drink in the *certain* condition. In the second trial, two customers approached the robot and one of them ordered a drink in the *uncertain* condition. After the practice trials, the ghosts were asked whether they felt confident with the task and whether anything in the display was unclear to them. In addition, the customers pointed out any problems through the chat connection. After the ghosts’ questions had been answered to their satisfaction, the twelve experimental trials were presented in system generated random order.

The introduction and calibration required about 20 min. The experimental session took about 25 to 30 min to complete so participants were scheduled for one hour sessions.

## Results

One participant was excluded from further analysis because the link between robot and control interface had broken down. We report data of 16 participants who completed 192 trials (excluding practice trials). The numbers of ordered, served and the trial-wise correctly served drinks are summarised in Table 1. The majority of drinks were served correctly indicating that the ghosts and their customers were able to establish a credible, successful dialogue. However, incorrectly identified speech utterances and whether the ghosts attended the information influenced the results, e.g. whether a group order was identified as one combined or two individual orders. In order to mitigate these effects, we adapted our analysis according to the data that were available to the ghosts. For example, if a customer ordered a juice but the ISR recognised two waters, we treated this as an order of two waters. If the ghost served the two waters, this drink order was scored as correctly served. Thus, we analysed how the ghosts responded to the speech hypotheses that they actually received from the ISR. It is important to note that we measured the actions of the participant with regard to the speech hypotheses and how they mitigated uncertainty rather than the quality of the ISR/ASR. The hypotheses could still update during the presentation and the ghosts could still make mistakes, e.g. by selecting the wrong drink compared to information that was available to them (see Table 1). Consequently, the served drinks differed from the scripted dialogues. In particular, the number of group orders was smaller than scheduled. The customers were aware of technical mishaps through their information screen.

**Table 1:** Number of scripted drink orders, drink orders detected by the sensors and number of correctly served drinks.

Type of order	Condition	Number of scripted orders	Number of detected orders	Number of correctly served orders	Ratio of correctly served orders
Individual order	*Certain*	96	124	115	93%
	*Uncertain*	96	133	99	74%
Group orders	*Certain*	32	19	18	95%
	*Uncertain*	32	20	14	70%
Total number of drinks	*Certain*	160	162	151	93%
	*Uncertain*	160	173	127	73%

A trial was scored as correct if the customers’ requests were served according to recogniser data. Only those drinks that were served in correct trials contributed to the number of correctly served drinks. Please note that a group order comprised of two drinks, thus 32 group orders contribute 64 drinks.

### Acknowledging new customers

In order to start an interaction, the ghosts had to identify new customers from the sensor data and communicate to them that they were ready to take their drink orders. We identified when the ghosts turned towards the control panel in order to select appropriate actions using the eye tracker data. The respective states of the indicators on the information panel are summarised in Table 2. For comparing the *certain* and *uncertain* condition, we summarised the continuous data in the *uncertain* condition into categories. The customers’ visibility and distance to the bar were categorised into false (no indication on display), true (at the bar) and approaching (intermediate state). The customers’ torso orientation was presented as an arrow in both conditions. However, if the sensor detected their torso orientation, the customers were more or less looking towards the bar. Thus, we summarised the data as customers looking towards the bar (true) if the sensor was able to determine their torso orientation and as false otherwise. In the vast majority of cases, the ghosts acknowledged new customers if they were visible, at the bar, and faced the bar. The ghosts did not wait for a verbal utterance from their customers. The first action that the ghosts selected for the acknowledegement are summarised in Table 3.

**Table 2:** States of the indicators when the ghosts acknowledged new customers.

Indicator	State	Certain		Uncertain	
		Number	Percent	Number	Percent
Customer visible	*True*	95	99%	96	100%
	*False*	1	1%	0	0%
Customer at bar	*True*	92	96%	89	93%
	*Approaching*	0	0%	7	7%
	*False*	4	4%	0	0%
Customer facing the bar	*True*	93	97%	95	99%
	*False*	3	3%	1	1%
Customer saying something	*True*	8	8%	33	34%
	*False*	88	92%	63	66%

**Table 3:** First action that the ghosts selected for acknowledging their new customers.

Selected action	Certain		Uncertain	
	Number	Percent	Number	Percent
Looking at customer	76	79%	70	73%
Verbal greeting	18	19%	22	23%
Other verbal utterance	1	1%	2	2%
No action	1	1%	2	2%

In ‘No action’ trials, the ghosts waited for the customers to place an order before initiating any action.

### Speech hypotheses

The response times (RT) were analysed only if the ghosts served the drinks correctly because an erroneous serving affected the time course of the entire trial. Out of 192 trials, we analysed the RTs of the 168 correctly served trials. We excluded three actions from the analysis that were correct but performed with very fast (26 and 105 ms) or extremely slow (210 s) RT after the first informative hypothesis. A total of 304 actions including drink servings and responses to questions about the menu were analysed.

In order to measure whether the ghosts used the early ISR hypotheses, their RT was measured from the onset of a hypothesis on the display until they shifted away towards the control panel. The time span approximates the time required for reading and understanding the hypotheses and for deciding whether to initiate a response action or whether to wait for more or better data. In addition to making that decision, the content of the hypotheses had to be memorised because re-reading them required the ghosts to turn back to the panel. Other measures would have included additional biases, e.g. the time until a click was performed includes biases from locating and positioning the mouse cursor on the control panel. In contrast, we relied on a shift of overt attention. The RTs were measured both from the onset of the first (*RT*_*first*_) and last (*RT*_*last*_) informative hypothesis. The first hypothesis (*RT*_*first*_) was defined as the first hypothesis that included sufficient information in order to respond correctly to the customer’s request. The most recent hypothesis that the ghosts attended before turning away was defined as the last hypothesis (*RT*_*last*_). In the *certain* condition, both metrics are often equal because only final hypotheses had been presented. However, in some cases the ISR marked two hypotheses as final. Also, if a ghost did not respond in the first instance, the customers repeated their utterance. In order to mitigate effects of individual differences in RT, a participant-wise *z*-score was computed and analysed (see Table 4).

**Table 4:** Summary of the number of cases, response times (RT), the corresponding *z*-scores (*z*-RT), the number of hypotheses and their confidence levels as a function of the type of request and the condition.

	Individual orders		Group orders		Questions about menu	
	*Certain*	*Uncertain*	*Certain*	*Uncertain*	*Certain*	*Uncertain*
**Number of cases**	115	98	17	14	28	32
**M of *RT***_***first***_ **in ms**	8249	8501	10992	20970	4909	5573
**SD of *RT***_***first***_ **in ms**	6561	6923	6677	20637	2821	6173
**M of *z-RT***_***first***_ **in ms**	0.00	0.06	0.41	1.32	-0.56	-0.49
**SD of *z-RT***_***first***_ **in ms**	0.91	0.93	0.64	1.63	0.42	0.81
**M of *RT***_***last***_ **in ms**	6191	3041	9423	5473	4838	3433
**SD of *RT***_***last***_ **in ms**	3493	3049	3369	5573	2835	2576
**M of *z-RT***_***last***_ **in ms**	0.36	-0.52	1.38	0.03	-0.03	-0.41
**SD of *z-RT***_***last***_ **in ms**	0.85	0.81	0.93	1.30	0.77	0.63
**M of # of hypotheses**	1.17	3.38	1.12	5.36	1.04	1.84
**SD of # of hypotheses**	0.46	1.46	0.49	3.32	0.19	0.95
**M of confidence level**	100	73	100	74	100	61
**SD of confidence level**		20		15		27

Only correct trials contributed to the measures. An individual order, a group order and a question were excluded for extremely fast/slow RT.

We used the statistical analysis program JASP [70]. We report the Bayes Factors from the respective ANOVAs and *t*-tests [71,72] that were obtained with a scale parameter of $\frac{1}{\sqrt{2}}$ for the Cauchy distribution serving as prior for the effect size [72]. In contrast to standard, frequentist statistical tests, Bayesian statistics also evaluates the amount of relative evidence in favour of the null hypothesis. In addition, we used mixed model analyses [73,74] as implemented in the lme4 package [75] of R. The denominator degrees of freedom and *p*-values were estimated with the Kenward-Roger approximation in the lmerTest [76] and pbkrtest packages [77]. We considered an effect to be statistically significant if both statistical methods agreed. The effect size of the equivalent *F*-test [78] or *t*-test [78] was computed with G*Power [79].

The mixed model analysed the type of request (*individual order*/*group order*/*question about menu*) with a Helmert contrast testing whether individual orders differed from group orders and whether the drink orders in general differed from questions about the menu. The condition (*certain*/*uncertain*) entered this analysis as a nested factor under the type of request. In the Bayesian analysis, the type of request and the condition entered as two main factors and their interaction (means were aggregated for the Bayesian *t*-test). In some analyses, the discrepancy between the frequentist *p*-value and the Bayes factor [80] was large and in the unexpected direction, i.e., the BayesFactor appears as a more lenient. This was attributed to a) the nested factor in the mixed model resulted in less balanced group sizes compared to the main effects in the Bayesian analysis, and b) under these circumstances, the Kenward-Roger approximation for *p*-values is more conservative than a standard ANOVA whose *p*-values are lower but probably underestimated [81,82]. The trials were scripted in pairs of a *certain* and a matched *uncertain* trial. However, due to errors and exclusions, the data set did not reflect the pairwise design. Thus, the *certain* and *uncertain* trials were analysed as independent samples.

Measured from the first hypothesis, there was a statistically significant effect of the type of request on *z-RT*_*first*_ [*F*(2,53.276) = 3.514, *p* = .037, *f* = 0.16, *BF*_*10*_ = 590.8]. The *BF* indicated that group orders required more time than individual orders [*BF*_*10*_ = 25.32] but the mixed model did not [*t*(24.13) = 1.149, *p* = .262, *d* = 0.22]. There was an unequivocal indication that the ghosts responded statistically significantly faster to questions than to drink orders [*t*(63.70) = 2.555, *p* = .013, *d* = 0.37, *BF*_*10*_ = 340848]. There was no statistically significant effect of the condition (*certain*/*uncertain*) on *z-RT*_*first*_ [*F*(3,26.175) = 0.626, *p* = .605, *f* = 0.08]. The Bayesian analysis revealed evidence against a main effect of the condition [*BF*_*10*_ = 0.313] and its interaction with the type of analysis [*BF*_*10*_ = 0.222].

There was a main effect of the type of request on the RT from the onset of the last hypothesis *z-RT*_*last*_ [*F*(2,34.37) = 13.453, *p*<.001, *f* = 0.30] in the mixed model analysis but not in the *BF* [*BF*_*10*_ = 2.591]. The contrasts showed that individual orders required statistically significantly shorter *z-RT*_*last*_ than group orders [*t*(19.67) = 4.437, *p*<.001, *d* = 0.86, *BF*_*10*_ = 193.8]. The mixed model indicated that questions were responded to faster than orders [*t*(44.71) = 4.378, *p*<.001, *d* = 0.63] but the *BF* did not provide clear evidence [*BF*_*10*_ = 1.606]. There was a statistically significant effect of the nested factor condition (*certain*/*uncertain*) on *z-RT*_*last*_ [*F*(1,19.62) = 21.336, *p*<.001, *f* = 0.46]. The *BF* indicated the corresponding main effect on *z-RT*_*last*_ [*BF*_*10*_ = 14.705] and interaction of the condition and the type of request [*BF*_*10*_ = 3.558]. The effect of the condition was tested for each type of request and revealed that *z-RT*_*last*_ in the *uncertain* condition were statistically significantly faster compared to the *certain* condition in individual orders [*t*(11.10) = 6.711, *p*<.001, *d* = 0.93, *BF*_*10*_ = 4.612e+6] and in group orders [*t*(35.18) = 4.330, *p*<.001, *d* = 1.62, *BF*_*10*_ = 5.246]. In contrast to orders, there was no such an effect in questions about the menu [*t*(32.17) = 1.511, *p* = .141, *d* = 0.39, *BF*_*10*_ = 1.829].

For further insights into how the ISR speech hypotheses were used, we analysed the *uncertain* trials with regard to the number of hypotheses and their confidence level. The type of request was analysed as above using a Helmert contrast in the mixed model. This showed a statistically significant effect on the number of required hypotheses [*F*(2,84.46) = 14.946, *p*<.001, *f* = 0.47]. The *BF* supported the corresponding main effect [*BF*_*10*_ = 14113]. Individual orders required statistically significantly fewer hypotheses than group orders in the mixed model [*t*(59.55) = 3.532, *p*<0.001, d = 1.02] but not with a Bayesian *t*-test [*BF*_*10*_ = 2.796]. Questions required statistically significantly fewer hypotheses than orders [*t*(87.86) = 5.459, *p*<.001, *d* = 1.10, *BF*_*10*_ = 934930]. The type of request had a statistically significant main effect on the confidence level of the last attended hypothesis [*F*(2,45.94) = 3.523, *p* = .038, *f* = 0.23, *BF*_*10*_ = 6.270]. There was no statistically significant difference between individual orders and group orders [*t*(35.12) = 0.015, *p* = .988, *BF*_*10*_ = 0.376] and there was no unequivocally conclusive evidence for such a difference between orders and questions [*t*(41.77) = 2.283, *p* = .028, *d* = 0.46, *BF*_*10*_ = 1.694].

### Fluency of the interaction

In order to estimate the fluency of the interaction, we analysed the time from the first visible customer action until the first drink had been served in each trial (see Table 5). In individual orders, we have to distinguish between drink orders that were preceded by a question and those that were not. The condition (*certain*/*uncertain*) and whether a question preceded placing the order entered the model as variables. There was a main effect of whether there was a question about the menu on the participantwise *z-scores* of serving time [*F*(1,44.12) = 14.810, *p*<.001, *f* = 0.34, *BF*_*10*_ = 330.5] indicating that the initial question prolonged the interaction duration. There was no main effect of the condition [*F*(1,7.709) = 1.644, *p* = .237, *BF*_*10*_ = 2.587]. The interaction of both factors was statistically significant [*F*(1,44.62) = 6.338, *p* = .015, *f* = 0.22, *BF*_*10*_ = 10.37]. This analysis indicated serving times were shorter in the *uncertain* compared to the *certain* condition if a question preceded the drink order. The group orders were not tested with respect to questions because only one group order was preceded by a question. This and another group order that was preceded by an individual order were excluded from this analysis. In the remaining 29 group orders, there was no effect of the condition on the *z-scores* of serving time [*F*(1,0.901) = 0.531, *p* = .610, *BF*_*10*_ = 0.828].

**Table 5:** Number of cases, serving time (RT), their participant-wise *z*-scores and the corresponding standard deviations from the first appearance of the customers until the first drink in the trial was served as a function of the type of request, preceding menu related questions and condition (*certain*/*uncertain*).

	Individual orders				Group orders	
	Preceding question		No question		No question	
	*Certain*	*Uncertain*	*Certain*	*Uncertain*	*Certain*	*Uncertain*
**Number of cases**	31	17	43	44	16	13
***RT* in ms**	61169	43262	29911	29220	30504	46130
***SD* in ms**	56001	24214	24754	8185	11605	26848
***z-RT***	0.72	0.14	-0.39	-0.27	-0.19	0.52
***SD* of *z-RT***	1.17	0.95	0.81	0.44	0.66	1.30

## Discussion

All ghost participants reported that they enjoyed taking part in the study. They were immersed in the setting and were highly engaged in the task. This allowed us to investigate spontaneous decisions regarding social behaviour in a service encounter. In order to develop a cognitively plausible interaction policy, we had to assume that humans expect other humans and artificial agents to behave like they themselves would. This is supported by the fact that typical mistakes in human-human communication can be attributed to so-called “egocentric” assumptions of the interlocutors [83], i.e. humans draw conclusions about the knowledge and understanding of others based on their own experience. Speakers failed to systematically use syntactic structures that are unambiguous to their listeners [84,85] and listeners systematically misinterpreted utterances with an egocentric bias [86]. Participants also used their own strategy for predicting the actions of others in social decision making, e.g., the prisoner’s dilemma or the give-some game [87–89]. The Bayesian rationale for this is that one’s own behaviour is a reasonable starting point for forming expectations [90]. In fact, one’s own behaviour is the only available data in anonymous one-shot social decisions [91]. The bar scenario is a brief, goal oriented social interaction whose participants typically do not know each other but they share prior knowledge about the situation. Thus, they would likely use their own behaviour for forming expectations about others in the same situation [88,92]. Assuming that others behave like yourself causes misunderstandings if the egocentric assumptions about others do not hold, e.g., if the speaker’s and the listener’s egocentric models differ [83]. We still suggest to model an artificial agent according to the participants’ (ghosts) own behavioural preferences. In this way, the artificial agent is similar to humans as it operates on egocentric assumptions. Thus, it could either produce the expected behaviour or commit a typically human mistake. However, whether the behavioural preferences of humans (e.g., as ghosts in the machine) are equal to their expectations on the behaviour of artificial agents remains an important question for further research.

### Ecological validity

The study was designed as similar as possible to a real human-robot interaction at the bar. The ghosts perceived their human customers through real robot sensors and responded with robot actions. They also experienced the time pressure of a real-time interaction with their customers. The experiments were resource intensive and thus, conducted with a small number of participants. However, they provided insights into the socially appropriate strategies for mitigating slow sensors, uncertainty and short response times.

The ecological validity was evaluated by comparing when the ghosts identified and how they addressed new customers in this study to previous findings. In real-life recordings of bars and in lab experiments [68] as well as in offline GiM studies [65,66], new customers were identified if they were close to the bar and looked towards the bar or bartender. Real bartenders and lab participants preferred to initiate a verbal interaction before their customers said something. We obtained very similar results with only one deviation, see Table 2. In about ⅓ of the interactions in the *uncertain* condition, the ghosts acknowledged their new customers after they said something. We attributed this to the faster pace of displaying early, uncertain speech hypotheses in the *uncertain* condition compared to the final hypotheses in the *certain* condition. Thus, the same RT could have preceded utterances in *certain* but succeeded them in *uncertain* trials. Also, accidental sounds from the environment may have triggered early speech hypotheses that were displayed in *uncertain* but not in *certain* trials. This can account for the small deviation from previous findings. Furthermore, we compared the type of action that the ghosts selected for acknowledging new customers to previous results. In this study, the ghosts looked at the new customers or greeted them verbally (Table 3) reflecting earlier results [66,68] in type and frequency of the selected actions. We concluded that the real-time GiM interface allowed the ghosts to interact with their customers socially appropriately. Thus, the collected data enable us to derive a cognitively plausible policy for using early, uncertain speech hypotheses in short social interactions with artificial agents.

### Use of speech hypotheses

The customers’ speech was the most important modality once the interaction had been initiated, [66]. The display of the speech hypotheses received the largest share of relative dwell time in this study (Table 6). Thus, responding to the users’ speech is highly important in service interaction with artificial agents. However, current ASRs require more time for fully analysing an utterance [31] than humans expect in conversation [2,5,6]. In order to use early ISR hypotheses in a psychologically plausible way, we have to identify when humans would rely on a partly processed utterance for initiating a response action. Humans recognise auditory utterances incrementally and provide grounding signals while it is still unfolding [22,28]. This serves as feedback to the speaker who can adapt her/his utterance and thereby ensure that the listener’s response action will be appropriate. In contrast to grounding, initiating a response requires that the listener has already committed to one interpretation of the utterance.

**Table 6:** Relative dwell times on indicators.

Indicator	Relative dwell time
Customer visible	0.11
Customer torso orientation	0.15
Customer at bar	0.12
Speech hypotheses	0.42
Elsewhere	0.21

The relative dwell time was computed by dividing the time span that a participant dwelled on each indicator by the summed dwell time on the information panel, and averaging across participants. This analysis is comparably coarse because the tracking accuracy was reduced as a result of the large head turns.

Utterance understanding and committing to one interpretation are rapid and elusive processes. In turn, it is difficult to identify when a human listener committed to a particular interpretation. In order to investigate when a human (ghost) initiates a response action, we extended the temporal gaps between the visual presentations of ISR hypotheses (time morphing). The delayed presentation of speech hypotheses provided the ghosts with sufficient time for reading, understanding and responding to the hypotheses. Time morphing combined with the GiM design enabled us to investigate whether, when and how humans use early, uncertain hypotheses with a slow motion variant of a typical ISR.

### Error cost

The RT from the onset of the first hypothesis (*RT*_*first*_) indicated that questions about the menu were responded to faster than drink orders. We attributed this difference to the potential error cost of the respective response action. Error cost is represented by the required efforts for rectifying the error and by the ‘loss of face’ associated with it [93]. The questions about the menu differed from the drink orders with regard to their error cost. Listing the short menu of three drinks was appropriate as a response to all questions about the menu and if new customers have just arrived at the bar. Thus, the error cost of listing the menu was small. In contrast, serving the wrong drink or unsolicitedly serving a drink was not appropriate. Furthermore, the bartending robot had no option of retracting or replacing a drink once the action had been initiated. Thus, the ghost would have had to apologise and negotiate an appropriate repair. Due to the restricted SRGS, the customers could not respond with the flexibility required for accepting or negotiating the ghost’s repair action. Consequently, rectifying an erroneous serving was never successful in this study and resulted in a breakdown of the interaction. If the ghosts experienced this, a breakdown added to their perceived error cost. Such errors occurred in 12.5% of the trials that were excluded from further analyses. It should be noted that it is difficult to detect and repair such breakdowns even without SRGS restrictions [94]. Thus, costly errors should be avoided and we discuss echo question as one measure to achieve this.

In general, RT can be regarded as a compound of a baseline and a decision time, cf. [95]. In our study, the baseline was required for understanding hypotheses and preparing a response. The decision time is the additional time needed before committing to an action. The ghosts attended the information panel during this time. By doing so, they could gain access to additional and/or better data, e.g. additional and more confident hypotheses in the *uncertain* condition.

Answering questions about the menu was associated with low potential error cost and with the fastest RT in our study. Thus, these RT are mainly composed of the baseline (reading and understanding hypotheses) and only little decision time. Compared to answering questions, the *RT*_*first*_ in drink servings were prolonged. The baseline was constant but the decision time was extended because the ghosts aimed for a higher level of confidence before initiating the serving due to the higher error cost of serving a drink compared to listing the menu. They achieved this higher confidence by a) trying to accumulate more evidence, i.e. additional and more confident speech hypotheses, and b) using echo questions (e.g., “A coke for you?”) for grounding their correct understanding in 62 out of 165 servings. Echo questions and the customer’s response increased the confidence in serving the correct drink. But choosing between serving the drink straight away and using an echo question added to the decision time. In contrast to servings, the ghosts never grounded whether they should list the drinks menu but initiated this action immediately even if the data were only suggestive. In sum, the ghosts required greater confidence before committing to a serving than to listing the menu. They achieved this by waiting for additional and more confident speech hypotheses or explicit grounding. Thus, the *RT*_*first*_ for drink orders were slower than for questions.

The differences in *RT*_*first*_ cannot be attributed to the ghosts’ efforts in identifying, planning or initiating the appropriate response action. The *RT*_*first*_ were measured from the onset of the first informative speech hypothesis. Thus, the *RT*_*first*_ do not include a period of time where the ghosts were unable to identify their customers’ speech act and the corresponding response action. However, they include reading the speech hypotheses. But reading the interface was comparable across the types of requests and cannot explain the observed RT pattern. The RTs were measured until the ghosts shifted their gaze from the information panel towards the control screen. The time required for finding the correct response button and navigating the mouse cursor were not included in the RT. Thus, differences in the spatial or visual saliency of the control buttons cannot account for the longer RT in drink orders than in questions. If anything, the drinks were represented by icons of bottles with a greater visual saliency than the text buttons. This would predict that listing menu required more time than serving a drink but the results showed the opposite pattern. Another possible explanation for the differences in *RT*_*first*_ is that the ghosts required more time for planning a serving than for listing the menu. This account also predicts that the time required for planning the serving of one drink (individual order) should be shorter than for two drinks (group order). However, there was no unequivocal statistical evidence in *RT*_*first*_ that indicated such a difference. Thus, the RT pattern cannot be explained by planning response actions.

The congruence of the modality of requests and responses could provide another explanation for the results. The modality of the response action was associated with fixed error cost (risk) in this study. The physical serving of a drink was associated with greater potential error cost than a verbal listing of the available drinks. But there is no general link between a modality and its potential error cost, e.g. verbal utterances in court can be very costly. However, there is a preference to respond to requests in the same modality [66]. The questions and their responses were congruent in using the verbal modality whereas drink orders were incongruent. If the ghosts were slower in initiating the response of incongruent than congruent request-response pairs, their RT should be slower in drink orders than in questions independently of the number and confidence of the speech hypotheses. But the results indicated that speech hypotheses were used strategically. In questions, the ghosts responded quickly and independently of whether the speech hypotheses were more or less confident. In drink orders, the ghosts showed a prolonged *RT*_*first*_ indicating that they accumulated evidence. But the *RT*_*last*_ measured from the last attended hypothesis showed that questions and drink orders required similar RTs once sufficient and confident data were available in the *uncertain* condition. This shows that the time for reading and understanding the hypotheses was similar across requests but the decision time was dependent on the error cost of the response, and the number and the confidence of the speech hypotheses.

In sum, the ghosts tried to accumulate more and more confident data if the potential error cost was higher. This resulted in longer decision times in drink orders compared to questions about the menu.

### Effect of uncertain hypotheses

The analysis of *RT*_*first*_ revealed no difference with regard to whether early, uncertain hypotheses were displayed or not. This could indicate that the participants a) fully relied on the early data, or b) they were not able to distinguish between different types of hypotheses. The results showed that the number of attended speech hypotheses differed significantly between the types of requests in the *uncertain* condition. This implies that the ghosts distinguished between confident and less confident hypotheses and that they used the hypotheses strategically depending on the potential error cost of their next action.

The strategic decision time can be estimated as the difference between *RT*_*first*_ and *RT*_*last*_. The questions about the menu provided the baseline for the analysis of the *RT*_*first*_ because of their low potential error cost and the fast RT. Similarly, the *RT*_*last*_ to questions can serve as baseline with minimal decision time. In questions, the *RT*_*first*_ and *RT*_*last*_ were often measured from the same hypothesis because the ghosts often responded to the first hypothesis that indicated a question. In turn, there was no statistically significant difference between questions in the *certain* and *uncertain* condition. Drink orders differed from questions with regard to the number of speech hypotheses and their RT pattern. The ghosts attended more speech hypotheses in drink orders than in questions in the *uncertain* condition. Also, *RT*_*last*_ were shorter in *uncertain* compared to *certain* trials in drink orders indicating that the ghosts’ decision was more dependent on the intermediate hypotheses. If they were available, the ghosts responded as fast as their baseline *RT*_*last*_, i.e. with minimal decision time. This indicates that the preceding time was used for accumulating evidence and deciding whether and when to commit to an interpretation. In the *uncertain* condition, intermediate hypotheses with similar content would support the ghosts’ confidence in the sensor data or provide counterevidence to their response plan. In the *certain* condition, the ghosts would have to rely on the absence of counterevidence. Thus, their *RT*_*last*_ was longer in the *certain* compared to the *uncertain* condition.

The group orders’ time line differed from that of individual orders. First, the utterances were slightly longer than in individual orders. This required additional processing by the ISR, triggered more speech hypotheses and in turn, the time morphing slowed the presentation significantly. Secondly, the ghosts had to memorise and check possible updates on two instead of one drink while preparing their response. Despite those differences, the *RT*_*last*_ were shorter when using early, uncertain hypotheses compared to using only final hypotheses. This advantage was similar to but smaller than in individual orders.

These results showed that the participants committed to response actions based on partly processed or anticipated content. Previous studies showed that incomplete material was used for anticipating the end of a turn [96], performing grounding gestures [22] and planning ahead [28] such that the response action is initiated just about the end of the speaker’s turn [2]. However, speech hypotheses differ from natural language as they do not have temporal information about the end of a turn, are delayed compared to an unfolding user utterance but appear in fast succession. In order to develop a psychologically plausible policy for using incremental speech hypotheses, we slowed down the presentation of incremental speech hypotheses. We demonstrated that the ghosts did not wait for a complete analysis of their customers’ utterances before initiating their response. If a request is plausible and the potential error cost is low, humans commit to their response. This suggests that humans would respond to utterances that they have not fully processed or only perceived partly. In order to fully understand the cognitive processes involved in using anticipated content, further experimental evidence is required. But our findings demonstrate that humans use early, uncertain ISR speech hypotheses and might expect others and artificial agents to do so.

### Benefit of using early hypotheses

There was a clear benefit of using early, uncertain ISR hypotheses. The time span between the first hypothesis and a response (*RT*_*first*_, see Table 4) was independent of whether the hypothesis was uncertain or final. But relative to the customers’ utterance, the ISR issues early, uncertain hypotheses about one to two seconds earlier than final hypotheses. Thus, the time span between the first and the final ISR hypothesis estimates how much faster a question was answered or a drink was served if uncertain hypotheses were available compared to when they were not.

There was no observable advantage of the *uncertain* condition beyond the time difference between the first and the final speech hypothesis. However, ghosts’ workload was larger in the *uncertain* compared to the *certain* condition. The ghosts had to read additional hypotheses, identify their confidence value, judge whether next hypotheses may change the current interpretation and select a response taking into account the available information and the potential error cost. These processes had to be repeated for each speech hypothesis, i.e. once in the *certain* condition and multiple times in the *uncertain* condition. This additional workload might have delayed the ghosts’ responses but it was necessary in order to benefit from integrating multiple hypotheses as converging evidence. It enabled the ghosts to pre-plan their response and to initiate it quickly once they had committed to an interpretation. Similarly, participants prepared their responses and waited for initiating them in interaction studies [28]. In this GiM study, this strategy was used specifically in drink orders that were associated with high error cost whereas questions were responded immediately. Thus, the increased workload in the *uncertain* compared to the *certain* condition might has hindered the ghosts from responding more timely. In turn, the temporal difference between the ISR’s first and the final speech hypothesis only approximates how much faster a question was answered in the *uncertain* compared to the *certain* condition, i.e. the net benefit of using uncertain hypotheses.

### Fluency of the interaction

In order to approximate the fluency of the interaction, we used the time span from the first visual information of the customers’ appearance until the first drink had been served [97]. This relatively coarse metric might be subject to influences including our own time morphing procedure. However, it approximates how fast the task of serving a drink had been achieved. Individual orders included trials with and without preceding questions about the menu (see Table 5). Without a preceding question, the time until the first serving was almost equal in the *certain* and *uncertain* conditions for individual and group orders. However, if a question preceded an individual drink order, there was a benefit of presenting early, uncertain hypotheses. This appears counterintuitive because questions contributed multiple additional hypotheses in the *uncertain* condition but only one in the *certain* condition. Thus, the time morphing should increase the required time in the *uncertain* condition but it did not. First, the ghosts responded quickly to the first indication of a question. Thus, they did not attend all hypotheses. Secondly, the first hypothesis in the *certain* condition was a final hypothesis. Thus, it was issued a few seconds after the comparable uncertain hypothesis. Thirdly, after responding to the question, the ghosts had just explained the menu to their customers and expected a drink order. In turn, they might have spent less time for preparing or deciding on their actions. This study does not allow to distinguish between these options but we can conclude that the *uncertain* condition benefited from the additional evidence about the customers’ utterances. That means that the time morphing and additional hypotheses did not reduce but improve the fluency of the dialogue. In a computational model, the evidence from preceding dialogue moves could contribute to the confidence of recognising current utterances and/or committing to an action.

### Policy for human-robot interaction

**Fig 5:**
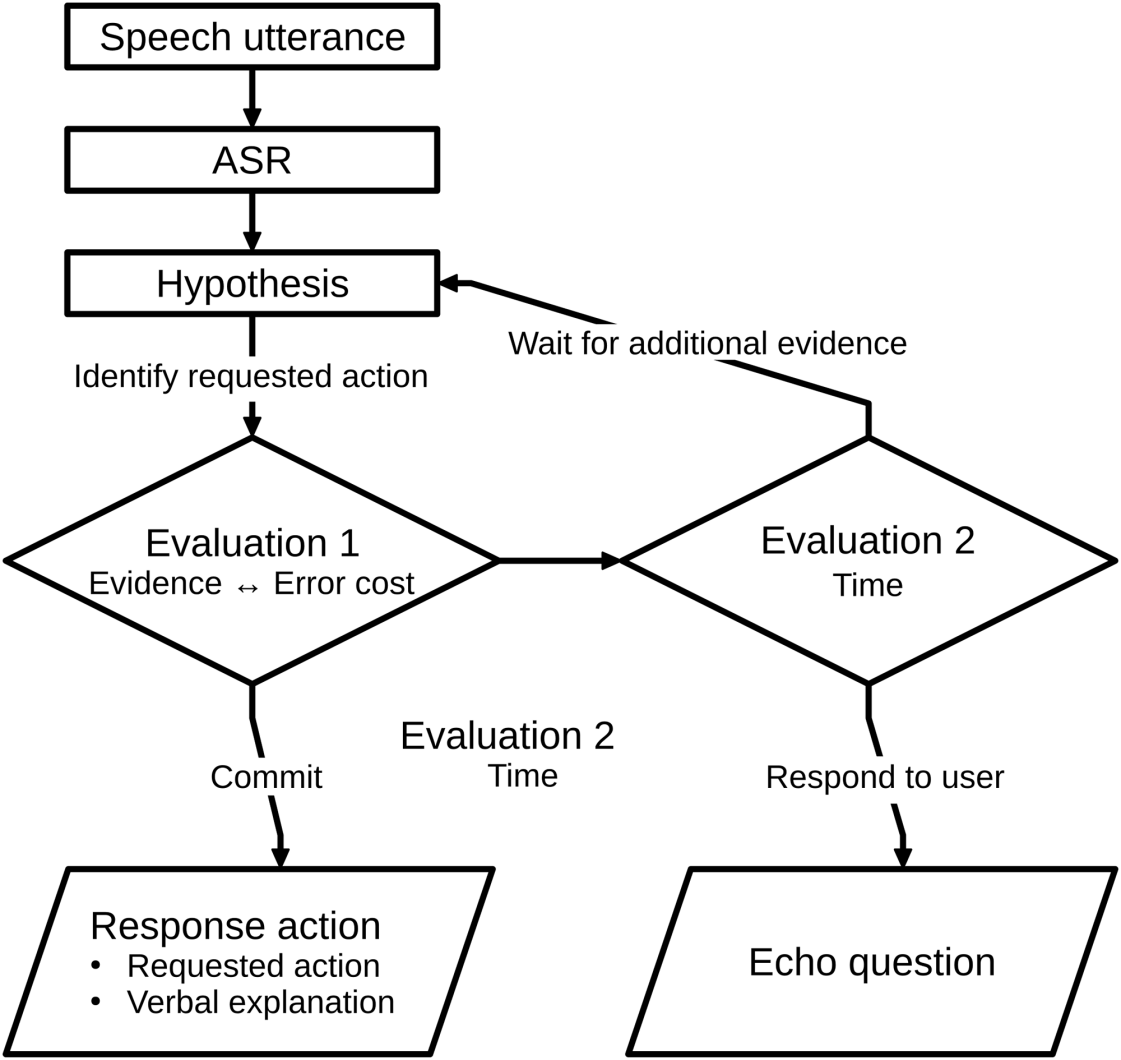
Flowchart of suggested human-robot interaction policy. The user’s speech utterance triggers the ASR that issues hypotheses about it. Comparing the error cost of the response action to the evidence decides whether to wait, ask an echo question or perform the action.

Typical interaction policies wait for the final hypothesis but this increases the response time to at least one second. As shown above, this delays turn-taking and disrupts the flow of the interaction. Furthermore, users might interpret the pause as signalling that the artificial agent has not understood their utterance, they requested something that is outside of the machine’s scope, or that they have made a mistake in using the machine. Thus, we investigated human mitigation strategies for interacting through slow and sometimes erroneous robot sensors. The results of this study enable us to outline a policy for human-robot interaction in Fig 5 that a) uses early, uncertain speech hypotheses in a human-like way, and thereby, b) responds more timely to its users, and c) acts socially appropriately. In particular, we outline a social strategy for resolving situations where the best response action is associated with high error cost, the sensor data are uncertain and the user expects a timely system response.

The user’s speech utterance triggers the ASR that produces speech hypotheses as it progresses with the analysis of the utterance. For each hypothesis, the dialogue manager identifies the response action that the user requested, e.g. [35,56]. The next step in our policy is to estimate the error cost that is associated with this action. Two factors contribute to this cost. First, the cost of repairing the performance of the response action if the user actually requested something else, i.e., the severity of the error. This could be approximated, e.g. as the number of actions or the time that is required for the repair. For example, verbally listing the menu is less severe than serving the wrong drink because the former is much easier to fix than the latter. Secondly, the cost of committing an error as such, i.e., the user might trust the system less irrespectively of the error’s severity. This could be approximated as a constant cost per error.

Deciding whether and when to perform the response action is the most important aspect of this social policy. This evaluation is performed in two steps and considers three criteria: a) the error cost explained above, b) the available evidence, i.e. the number of converging hypotheses, their confidence value, and the dialogue state, and c) the time that has passed since the user’s utterance. The first step (Evaluation 1 in Fig 5) compares the error cost and the evidence. If the error cost is very low, the agent should execute the response action. If the error cost is higher, the number of converging speech hypotheses and their confidence level have to be assessed. In addition, the preceding dialogue has to be considered. For example, whether the user utterance is a second part of an adjacency pair, such as an answer to a question or the confirmation of a preceding statement [1,98]. If the available evidence is sufficient compared to the error cost, the agent executes the response action. Otherwise, the time constraint is assessed in a second step (Evaluation 2 in Fig 5). The evaluation will only arrive at this step, if the error cost is high and the evidence insufficient. If the timeliness constraints force the agent to perform a response now, the agent uses an echo question or statement. Otherwise, the agent can afford to wait for additional or better evidence with upcoming hypotheses until either new evidence is available or a response is due. This policy outlines when and how to proceed in the dialogue. Our knowledge about timing a response is very good but the precise statistics with regard to the quality of evidence from speech hypotheses are subject to future research. Relative to the GiM study, both evaluations take place in the decision time where the participants had to decide whether to commit to the response action, ask a question, or wait for more evidence.

Echo questions are a socially appropriate way of gathering additional evidence and avoiding errors, e.g. “A coke for you?”, cf. [66]. In addition to questions, the ghosts explicitly grounded [27] their understanding in about 37% of the drink orders prior to the serving with echo statements, “Here is your coke.”. These echo utterances repeat the key content of the user’s utterance [99] and, if the repetition was erroneous, expose misunderstandings quickly by providing the user an opportunity for corrections [42,44]. An echo question does not replace the response action but works towards more certainty with regard to it. For example, in Clark and Krych’s study [22], hearers poised their hand above the suspected target item before performing the response action and grasping the target. Signalling agreement to an echo is typically achieved through small gestures (e.g., nodding) or short statements (e.g., “yes”) or similar. In contrast, disagreements tend to be mitigated, delayed and involve more verbal material [6,100] and thus, are easy to identify from recogniser data. Furthermore, disagreements most likely repeat the correct key content, e.g. “No, a water, please.” However, these questions do not fulfil the requirements of minimal repetition and minimising the number of turns. Thus, they are in conflict with many reward functions used for machine learning [52]. But using adaptive grounding [42] and social interaction strategies [97] improved the agent’s subjective ratings, dialogue success and surprisingly, the increased number of turns reduces the dialogue duration. In sum, the explicit grounding strategy is socially appropriate, occurs frequently, is associated with minimal error cost and allows timely responses.

The social policy also requires that the response includes at least some action in the modality of the user’s request. For example, serving of the drink in response to a drink order is sufficient from a functional perspective. However, our results suggest that performing a motor action is not enough but that the user expects a verbal response as well. Thus, at least a short verbal response is required, e.g. a short confirmation such as "Here you are." This serves the users’ expectations that a request receives a response in the same modality, i.e. a verbal request receives a verbal feedback. Maintaining the same modality reflects a human tendency to imitate conversation partners. For example, speakers adapt their choice of words [101,102], their pronunciation [103], prosodic features [104] but also their posture [105] to each other. This co-adaptation improves different metrics of perceiving interactions and rapport [102,105,106].

In sum, this social policy enables the robot to respond more timely, socially appropriately and in the user’s modality. The timely, human-like responses enhance the immediacy of the interaction. It is still difficult for the robot to initiate a response within a time window of 250 ms before and after the user’s end of turn given the delays in speech recognition, speech production and the robot actuators. However, this policy uses the early speech hypotheses and, as we have argued above, can improve the response speed in many cases. By relying on socially appropriate strategies and incorporating psychological principles, the robot’s behaviour satisfies the users’ expectations on timing, manner and response selection. Thus, the artificial agent responds more timely and handles uncertainty more human-like.

### Limitations of the study

The bar scenario involves multiple customers in front of the robot that may engage in different activities such as chatting or placing a drink order and challenges the robot to correctly interpret human social behaviour. This complex scenario is restricted by a bar script [107] of typical customer-staff interactions. This might have enabled the ghosts to anticipate the next moves of their customers even without sensor data. In addition, the SRGS was limited such that the speech recognition was biased towards the correct content. Thus, the ghosts could rely on both their knowledge about the script and the SRGS for improving their performance. But enhancing perception by boosting or pre-activating also reflects basic principles in human cognition [108,109] and has been successfully used in speech recognition, e.g. [48]. Thus, our results can generalise to other situations, especially to short-term dialogues that are governed by a script.

Using the ISR and other recognisers under real-life conditions resulted in greater statistical noise than artificial data and some unexpected outputs. We mitigated this by adapting our analyses accordingly. Therefore only a small number of group orders could be analysed. However, the real-life ISR data highlighted how early speech hypotheses can contribute to create immediacy with existing technology. Even though the sensor data provide only a coarse approximation of human anticipation, our insights allow targeted experimental investigations, e.g. with respect to the confidence in human anticipation, sensor data in HMI and the cost of erroneously performing an action.

## Conclusions

The new interactive Ghost-in-the-Machine (GiM) design enabled us to investigate incrementality in human speech recognition and its role in social human-robot interaction. We showed the validity of the new design by comparing how the participants (ghosts) initiated the interaction with their customers to the behaviour real-life recordings [68]. The ghosts used a sophisticated method for accounting for a) the sensors’ confidence, b) the number of presented hypotheses, and c) the potential error cost of an action when selecting and initiating an action. We have argued that if humans interacted with an artificial agent, they would expect it to respond in a similar way, i.e. it should account for these factors. Existing models of processing incremental speech hypotheses, e.g. [61], would have to include the number of hypotheses and the error cost of the requested action. Such an extension makes the evaluation of speech hypotheses similar to evidence evaluation in decision theory frameworks that account of expected losses and the confidence in the evidence.

We identified the timeliness of the agent’s responses and their social appropriateness as important benchmarks. In natural interaction, human interlocutors prepare their response [2] and ground their understanding [22] while utterances unfold. They typically avoid overlapping speech [1] and time their responses very precisely within 250 ms before and after the turn end [2,3]. Thus, humans expect socially competent artificial agents to adhere to similar constraints on timeliness. But agents typically respond with a delay because they start planning only after the final analysis of the user’s utterance was available. In addition to the ASR, robot actuators and/or text-to-speech add further delays. In turn, users might mistake these gaps in the interaction as signalling an error. Thus, responding timely is difficult, especially if the error cost of the action is high, the certainty of the sensor data low and the user expects an immediate response. We devised a social response strategy that implements human social strategies for responding timely and socially appropriate while minimising errors.

The social interaction policy is based on the results of our online study. We found that the ghosts initiated actions while the ISR still analysed the user’s utterance, i.e. while speech hypotheses were still uncertain and the content was unfolding on the display. But the ghosts only initiated actions that were associated with low potential error cost (e.g., listing the menu). In turn, the customer’s request was responded faster if the ghosts had access to uncertain speech hypotheses because the ISR provided them earlier than the full analysis. Thus, the policy relies on speech hypotheses and first evaluates the evidence (confidence of the speech hypotheses, number of preceding, converging hypotheses) and the error cost of the response action. If an error would have been more costly (e.g., serving the wrong drink), the ghosts waited for additional and more confident speech hypotheses before initiating their response. However, if the ghosts waited for too long, this would have delayed their response. Thus, the second evaluation considers the response time. If the ghosts perceived a pressure to respond but a lack of evidence, they used echo questions (e.g., “A juice for you?”). This is a form of explicit grounding that a) enabled the ghosts to respond quickly with an action that was associated with low error cost, b) elicit the correct information, and c) maintain a socially appropriate interaction. Furthermore, these questions guide users into a predictable response. Agreeing to an echo question is typically a very brief utterance (e.g., “Yes.”) or a small gesture (e.g., nodding). In contrast, corrections require more verbal material and typically include the correct key content, e.g. “No, I would like a juice, please.” An agent can distinguish between agreement and objection based on the user’s utterance length and could deliver timely responses.

To summarise, this study provides evidence that humans use early, uncertain hypotheses not only for pre-planning but also for initiating response actions. Furthermore, we provided a psychologically plausible policy for creating immediacy in interactions with socially competent artificial agents by using early, uncertain speech hypotheses, their confidence level and the potential error cost as well as grounding actions.

## Supporting information

S1 File Customer script. The file lists the scripts that the customers used for ordering drinks.
(PDF)

S2 File Data set speech hypotheses. The data file includes the response times of the ghost participants from the first and last hypothesis in ms and *z*-scores. The file includes the type of response and the condition. Cases with wrong responses and the three excluded cases are marked.
(DAT)

S3 File Time to first serving. The file includes the type of serving (individual or group), whether the serving was preceded by questions, the condition and the serving time in ms and *z*-scores.
(DAT)

S1 Video Display of speech hypotheses in real-time. The video shows the speed of incremental speech hypotheses without time morphing.
(AVI)

S2 Video Display of speech hypotheses with time-morphing. The video shows the same example as S2 video as it was presented in the study including the eye tracking overlay of the ghost participant.
(AVI)
