# Supplementary material for: Confidence in uncertainty: Error cost and commitment in early speech hypotheses
Source: PLoS One. 2018 Aug 1;13(8):e0201516. doi: 10.1371/journal.pone.0201516 (PMC6070273; doi:10.1371/journal.pone.0201516)
Supplement: S1 File — The file lists the scripts that the customers used for ordering drinks. (PDF) [file pone.0201516.s001.pdf]

Scripts

Practice

One individual order

Customer 1: [approaches bar]

Customer 1: I would like a water, please. [Ich hätte gern ein Wasser, bitte.]

Robot: ….

Customer 1: Thank you. [Dankeschön.]

Individual order with a bystander

Customer 2: [approaches bar]

Customer 1: [bystander]

Customer 2: I would like a coke, please. [Ich hätte gern eine Cola, bitte.]

Robot: ….

Customer 2: Thank you. [Dankeschön.]

Experiment

Individual order with a bystander – version 1

Customer 1: [approaches bar]

Customer 2: [bystander]

Customer 1: I would like a DRINK, please [Ich hätte gern ein DRINK, bitte.]

Robot: ….

Customer 1: Thank you. [Dankeschön.]

Individual order with a bystander – version 2

Customer 2: [approaches bar]

Customer 1: [bystander]

Customer 2: Do you have a DRINK? [Haben Sie ein DRINK?]

Robot: ….

Customer 2: Okay, I'll have a DRINK. [Dann nehme ich ein DRINK.]

Robot: ….

Customer 2: Thank you. [Dankeschön.]

Two individual orders – version 1

Customer 1 & 2: [approach bar]

Customer 1: What kind of drinks do you have? [Was für Getränke haben Sie?]

Robot: ….

Customer 1: A DRINK, please. [Ein DRINK, bitte.]

Robot: ….

Customer 1: Thank you. [Dankeschön.]

Customer 2: I like a DRINK, please. [Ich möchte ein DRINK, bitte.]

Robot: ….

Customer 2: Thanks. [Danke.]

Two individual orders – version 2

Customer 1 & 2: [approach bar]

Customer 2: I take a DRINK, please. [Ich nehme ein DRINK, bitte.]

Robot: ….

Customer 2: Thank you. [Dankeschön.]

Customer 1: I would like a DRINK, please. [Ich möchte ein DRINK 1, bitte.]

Robot: ….

Customer 1: Thanks. [Danke.]

Group orders – version 1

Customer 1 & 2: [approach bar]

Customer 1: A DRINK and I'll take a DRINK, please. [Einen DRINK und ich nehme ein DRINK, bitte.]

Robot: ….

Customer 1: Thanks. [Danke.]

Group orders – version 2

Customer 1 & 2: [approach bar]

Customer 2: I'd have a DRINK and DRINK, please. [Ich hätte gern ein DRINK und DRINK, bitte.]

Robot: ….

Customer 2: Thanks. [Danke.]
